# Cyclo­hexyl­ammonium nitrate

**DOI:** 10.1107/S1600536814002244

**Published:** 2014-02-05

**Authors:** Abdulaziz A. Bagabas, Mohamed F. A. Aboud, Ahsan M. Shemsi, Emad S. Addurihem, Zeid A. Al-Othman, C. S. Chidan Kumar, Hoong-Kun Fun

**Affiliations:** a Petrochemicals Research Institute, King Abdulaziz City for Science and Technology, Riyadh 11442, Saudi Arabia; b Sustainable Energy Technologies (SET) Center, College of Engineering, King Saud University, PO Box 800, Riyadh 11421, Saudi Arabia; c Center for Environment and Water, King Fahd University of Petroleum and Minerals, Dhahran 31261, Saudi Arabia; d Chemistry Department, King Saud University, Riyadh 11451, Saudi Arabia; e X-ray Crystallography Unit, School of Physics, Universiti Sains Malaysia, 11800 USM, Penang, Malaysia; f Department of Pharmaceutical Chemistry, College of Pharmacy, King Saud University, PO Box 2457, Riaydh 11451, Saudi Arabia

## Abstract

In the title salt, C_6_H_14_N^+^·NO_3_^−^, the cyclo­hexyl ring adopts a chair conformation. The ammonium group occupies an equatorial position and the crystal struture is stabilized by inter­molecular N—H⋯O hydrogen-bonding inter­actions, resulting in a three-dimensional network.

## Related literature   

For the Brønsted–Lowry basicity behavior of cyclo­hexyl­amine, see: Solomons (1996 ▶). For the preparation of salts of anions and complex anions with cyclo­hexyl primary ammonium cations, see: Jones *et al.* (1998 ▶); Kolev *et al.* (2007 ▶); Lock *et al.* (1981 ▶); Muthamizhchelvan *et al.* (2005 ▶); Wang *et al.* (2005 ▶); Yun *et al.* (2004 ▶). For precautions relating to the reaction of cyclo­hexyl­amine with strong acids or oxidizing agents, see: Chang (2008 ▶); Patnaik (2007 ▶). For the structures of other cyclo­hexyle­ammonium salts, see: Shimada *et al.* (1955 ▶); Smith *et al.* (1994 ▶); Odendal *et al.* (2010 ▶). For ring conformations and ring puckering analysis, see: Cremer & Pople (1975 ▶). For reference bond lengths, see: Allen *et al.* (1987 ▶).
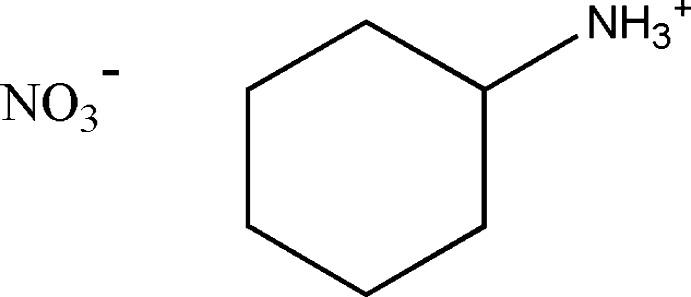


## Experimental   

### 

#### Crystal data  

C_6_H_14_N^+^·NO_3_^−^
*M* *_r_* = 162.19
Monoclinic, 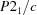

*a* = 8.9322 (9) Å
*b* = 9.9010 (9) Å
*c* = 10.3951 (10) Å
β = 103.866 (2)°
*V* = 892.53 (15) Å^3^
*Z* = 4
Mo *K*α radiation
μ = 0.10 mm^−1^
*T* = 294 K
0.39 × 0.15 × 0.14 mm

#### Data collection  

Bruker APEXII CCD diffractometer
Absorption correction: multi-scan (*SADABS*; Sheldrick, 1996 ▶) *T*_min_ = 0.964, *T*_max_ = 0.987
2214 measured reflections
2214 independent reflections
1750 reflections with *I* > 2σ(*I*)

#### Refinement  

*R*[*F* ^2^ > 2σ(*F* ^2^)] = 0.040
*wR*(*F* ^2^) = 0.121
*S* = 1.09
2214 reflections
101 parameters
H-atom parameters constrained
Δρ_max_ = 0.15 e Å^−3^
Δρ_min_ = −0.15 e Å^−3^

### 

Data collection: *APEX2* (Bruker, 2008 ▶); cell refinement: *SAINT* (Bruker, 2008 ▶); data reduction: *SAINT*; program(s) used to solve structure: *SHELXS97* (Sheldrick, 2008 ▶); program(s) used to refine structure: *SHELXL97* (Sheldrick, 2008 ▶); molecular graphics: *SHELXTL* (Sheldrick, 2008 ▶); software used to prepare material for publication: *SHELXTL* and *PLATON* (Spek, 2009 ▶).

## Supplementary Material

Crystal structure: contains datablock(s) global, I. DOI: 10.1107/S1600536814002244/sj5386sup1.cif

Structure factors: contains datablock(s) I. DOI: 10.1107/S1600536814002244/sj5386Isup2.hkl

Supporting information file. DOI: 10.1107/S1600536814002244/sj5386Isup3.cml

CCDC reference:

Additional supporting information:  crystallographic information; 3D view; checkCIF report

## Figures and Tables

**Table 1:** Hydrogen-bond geometry (Å, °)

*D*—H⋯*A*	*D*—H	H⋯*A*	*D*⋯*A*	*D*—H⋯*A*
N1—H1⋯O1^i^	0.97	1.89	2.8553 (14)	172
N1—H2⋯O3^ii^	0.94	1.97	2.9074 (15)	172
N1—H3⋯O1^iii^	0.85	2.24	2.9880 (15)	148
N1—H3⋯O3^iii^	0.85	2.28	3.0689 (15)	155

Symmetry codes: (i) 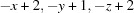; (ii) 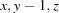; (iii) 
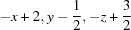
.

**Fig. 1.**
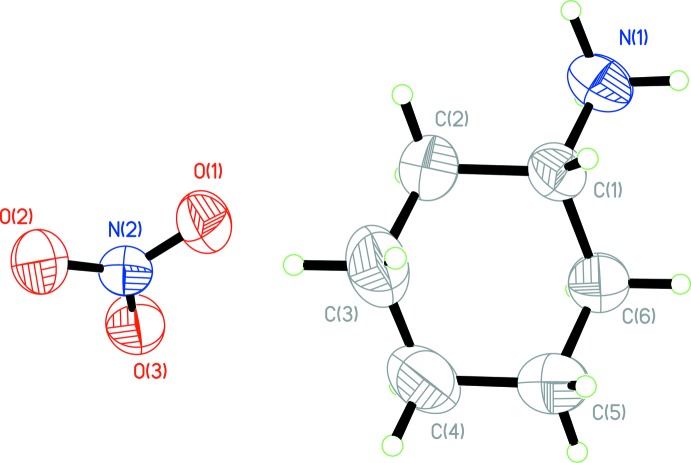
Molecular structure of the compound, with atom labels and 50% probability displacement ellipsoids for the non-H atoms.

**Fig. 2.**
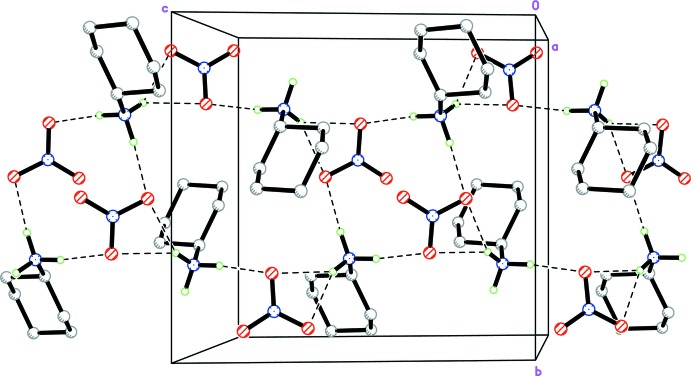
Crystal packing of the title compound, showing the hydrogen bonding interactions as dashed lines.

## supplementary crystallographic information

### 1. Comment

The title compound C_6_H_11_NH_3_^+^NO_3_^-^ was obtained as the unexpected by-product of the reaction of metal (M) nitrate salts (metal = Mg^2+^, Al^3+^, Cr^3+^, Mn^2+^, Fe^3+^, Co^2+^, Ni^2+^, Cu^2+^, Zn^2+^, or Cd^2+^) with cyclohexylamine (CHA) in either aqueous or ethanolic media. It was expected that CHA would coordinate to the M cations due to its Lewis basicity. However, metal oxides or hydroxides were formed along with C_6_H_11_NH_3_^+^NO_3_^-^, which reflects the Brønsted-Lowry basicity of CHA (pK_b_ = 3.36, Solomons, 1996). This base strength makes CHA suitable for the preparation of several salts of anions and complex anions through the formation of the primary ammonium cation (C_6_H_11_NH_3_^+^) (Jones *et al.*, 1998; Kolev *et al.*, 2007; Lock *et al.*, 1981; Muthamizhchelvan *et al.*, 2005; Shimada *et al.*, 1955; Smith *et al.*, 1994; Wang *et al.*, 2005; Yun *et al.*, 2004). This Brønsted-Lowry behavior was responsible for the formation of the present compound, (I) (Fig. 1), which is dangerous to prepare from a direct reaction between CHA and nitric acid (HNO_3_) because CHA reacts violently with strong acids or oxidizing agents and may cause fire and explosion (Chang, 2008; Patnaik, 2007).

The asymmetric unit of the title compound contains one cyclohexylammonium cation (C1—C6/N1) and one nitrate anion (N2/O1—O3). The cyclohexane ring adopts a chair conformation, with puckering parameters: Q = 0.5668 (17) Å, θ = 179.29 (17)°, and φ = 276 (21)° (Cremer & Pople, 1975). The ammonium functional group is at an equatorial position to minimize 1,3 and 1,5 di-axial interactions. The bond lengths (Allen *et al.*, 1987) and bond angles are in the normal ranges and are comparable with those reported earlier for similar compounds (Shimada *et al.*, 1955; Smith *et al.*, 1994; Odendal *et al.*, 2010). Each proton of the ammonium group is hydrogen-bonded to two oxygen atoms of the nitrate ion. These intermolecular N–H···O hydrogen bonds (Table 2) generate a three-dimensional network (Fig. 2).

### 2. Experimental

The title compound C_6_H_11_NH_3_^+^NO_3_^-^ was obtained as a by-product upon combining 60 ml, 0.5 *M* of metal nitrate (metal = Mg^2+^, Al^3+^, Cr^3+^, Mn^2+^, Fe^3+^, Co^2+^, Ni^2+^, Cu^2+^, Zn^2+^, or Cd^2+^) with 20 ml, 3.0 *M* (for divalent metal) or 4.5 *M* (for trivalent metal) CHA in aqueous or ethanolic media. Depending on the identity of M, a metal hydroxide or oxide was precipitated. Filtering this precipitate resulted in a clear filtrate, which upon the gradual evaporation of the solvent at room temperature resulted in the deposition of beautiful, colorless crystals of HCHA^+^NO_3_^-^. The chemical composition of these crystals was determined by C, H, N elemental microanalysis: (%C: 44.47 exp; 44.43 cal.), (%H: 8.70 exp.; 8.72 cal.), (%N: 17.26 exp.; 17.28 cal.), and (%O: 29.61 exp.; 29.59 cal.).

### 3. Refinement

The nitrogen-bound H-atoms were located in a difference Fourier map and were fixed at their found positions (N–H = 0.8498, 0.9440 and 0.9724 Å), with *U*_iso_(H) = 1.2 *U*_eq_(N). Other H atoms were positioned geometrically (C=H 0.97–0.98 Å) and refined using a riding model with *U*_iso_(H) = 1.2 *U*_eq_(C)

### Crystal data

**Table:**

C_6_H_14_N^+^·NO_3_^−^	*F*(000) = 352
*M**_r_* = 162.19	*D*_x_ = 1.207 Mg m^−^^3^
Monoclinic, *P*2_1_/*c*	Mo *K*α radiation, λ = 0.71073 Å
Hall symbol: -P 2ybc	Cell parameters from 11857 reflections
*a* = 8.9322 (9) Å	θ = 2.4–28.3°
*b* = 9.9010 (9) Å	µ = 0.10 mm^−^^1^
*c* = 10.3951 (10) Å	*T* = 294 K
β = 103.866 (2)°	Block, colorless
*V* = 892.53 (15) Å^3^	0.39 × 0.15 × 0.14 mm
*Z* = 4	

### Data collection

**Table:**

Bruker APEXII CCD diffractometer	2214 independent reflections
Radiation source: fine-focus sealed tube	1750 reflections with *I* > 2σ(*I*)
Graphite monochromator	*R*_int_ = 0.000
φ and ω scans	θ_max_ = 28.3°, θ_min_ = 2.4°
Absorption correction: multi-scan (*SADABS*; Sheldrick, 1996)	*h* = −11→11
*T*_min_ = 0.964, *T*_max_ = 0.987	*k* = 0→13
2214 measured reflections	*l* = 0→13

### Refinement

**Table:**

Refinement on *F*^2^	Secondary atom site location: difference Fourier map
Least-squares matrix: full	Hydrogen site location: inferred from neighbouring sites
*R*[*F*^2^ > 2σ(*F*^2^)] = 0.040	H-atom parameters constrained
*wR*(*F*^2^) = 0.121	*w* = 1/[σ^2^(*F*_o_^2^) + (0.0584*P*)^2^ + 0.0786*P*] where *P* = (*F*_o_^2^ + 2*F*_c_^2^)/3
*S* = 1.09	(Δ/σ)_max_ = 0.005
2214 reflections	Δρ_max_ = 0.15 e Å^−^^3^
101 parameters	Δρ_min_ = −0.15 e Å^−^^3^
0 restraints	Extinction correction: *SHELXL97* (Sheldrick, 2008), Fc^*^=kFc[1+0.001xFc^2^λ^3^/sin(2θ)]^-1/4^
Primary atom site location: structure-invariant direct methods	Extinction coefficient: 0.042 (6)

### Special details

**Table:**

Geometry. All e.s.d.'s (except the e.s.d. in the dihedral angle between two l.s. planes) are estimated using the full covariance matrix. The cell e.s.d.'s are taken into account individually in the estimation of e.s.d.'s in distances, angles and torsion angles; correlations between e.s.d.'s in cell parameters are only used when they are defined by crystal symmetry. An approximate (isotropic) treatment of cell e.s.d.'s is used for estimating e.s.d.'s involving l.s. planes.
Refinement. Refinement of *F*^2^ against ALL reflections. The weighted *R*-factor *wR* and goodness of fit *S* are based on *F*^2^, conventional *R*-factors *R* are based on *F*, with *F* set to zero for negative *F*^2^. The threshold expression of *F*^2^ > σ(*F*^2^) is used only for calculating *R*-factors(gt) *etc*. and is not relevant to the choice of reflections for refinement. *R*-factors based on *F*^2^ are statistically about twice as large as those based on *F*, and *R*- factors based on ALL data will be even larger.

### Fractional atomic coordinates and isotropic or equivalent isotropic displacement parameters (Å^2^)

**Table:**

	*x*	*y*	*z*	*U*_iso_*/*U*_eq_	
N1	0.94737 (12)	0.24027 (11)	0.84734 (10)	0.0591 (3)	
H1	1.0040	0.2396	0.9397	0.071*	
H2	0.9334	0.1504	0.8165	0.071*	
H3	1.0001	0.2818	0.8017	0.071*	
C1	0.79115 (13)	0.30167 (11)	0.83331 (11)	0.0504 (3)	
H4	0.7312	0.2428	0.8779	0.060*	
C2	0.80481 (15)	0.43882 (13)	0.89905 (13)	0.0619 (3)	
H5	0.8701	0.4967	0.8605	0.074*	
H6	0.8523	0.4293	0.9928	0.074*	
C3	0.64691 (18)	0.50274 (15)	0.88097 (18)	0.0806 (4)	
H7	0.6580	0.5927	0.9190	0.097*	
H8	0.5855	0.4495	0.9276	0.097*	
C4	0.56468 (19)	0.51139 (15)	0.7354 (2)	0.0887 (5)	
H9	0.4622	0.5482	0.7270	0.106*	
H10	0.6208	0.5721	0.6906	0.106*	
C5	0.55222 (17)	0.37454 (16)	0.67001 (17)	0.0833 (5)	
H11	0.5052	0.3842	0.5762	0.100*	
H12	0.4864	0.3168	0.7081	0.100*	
C6	0.70975 (16)	0.30923 (13)	0.68820 (12)	0.0638 (3)	
H13	0.6979	0.2189	0.6509	0.077*	
H14	0.7716	0.3614	0.6412	0.077*	
O1	0.90382 (12)	0.78324 (9)	0.87802 (9)	0.0694 (3)	
O2	0.82965 (10)	0.96820 (10)	0.95391 (9)	0.0681 (3)	
O3	0.87733 (12)	0.96351 (10)	0.75996 (9)	0.0729 (3)	
N2	0.86827 (10)	0.90604 (10)	0.86507 (9)	0.0529 (3)	

### Atomic displacement parameters (Å^2^)

**Table:**

	*U*^11^	*U*^22^	*U*^33^	*U*^12^	*U*^13^	*U*^23^
N1	0.0641 (6)	0.0561 (5)	0.0586 (6)	0.0105 (4)	0.0177 (4)	0.0154 (4)
C1	0.0543 (6)	0.0449 (5)	0.0521 (6)	0.0010 (4)	0.0129 (5)	0.0065 (4)
C2	0.0651 (7)	0.0546 (7)	0.0635 (7)	−0.0030 (5)	0.0108 (6)	−0.0057 (5)
C3	0.0777 (9)	0.0615 (8)	0.1048 (12)	0.0089 (7)	0.0265 (9)	−0.0157 (8)
C4	0.0674 (8)	0.0606 (8)	0.1274 (15)	0.0139 (7)	0.0022 (9)	0.0051 (8)
C5	0.0720 (8)	0.0705 (9)	0.0906 (10)	0.0057 (7)	−0.0135 (7)	0.0017 (8)
C6	0.0754 (8)	0.0569 (7)	0.0535 (7)	0.0052 (6)	0.0041 (6)	0.0008 (5)
O1	0.0868 (6)	0.0492 (5)	0.0696 (6)	0.0075 (4)	0.0139 (5)	0.0001 (4)
O2	0.0688 (5)	0.0736 (6)	0.0650 (5)	0.0105 (4)	0.0221 (4)	−0.0094 (4)
O3	0.0975 (7)	0.0663 (6)	0.0549 (5)	0.0129 (5)	0.0181 (5)	0.0090 (4)
N2	0.0479 (5)	0.0538 (5)	0.0536 (5)	0.0040 (4)	0.0051 (4)	−0.0018 (4)

### Geometric parameters (Å, º)

**Table:**

N1—C1	1.4968 (15)	C3—H8	0.9700
N1—H1	0.9724	C4—C5	1.508 (2)
N1—H2	0.9440	C4—H9	0.9700
N1—H3	0.8498	C4—H10	0.9700
C1—C6	1.5112 (16)	C5—C6	1.519 (2)
C1—C2	1.5119 (17)	C5—H11	0.9700
C1—H4	0.9800	C5—H12	0.9700
C2—C3	1.5159 (19)	C6—H13	0.9700
C2—H5	0.9700	C6—H14	0.9700
C2—H6	0.9700	O1—N2	1.2556 (13)
C3—C4	1.518 (3)	O2—N2	1.2261 (12)
C3—H7	0.9700	O3—N2	1.2516 (13)
			
C1—N1—H1	110.6	H7—C3—H8	108.0
C1—N1—H2	107.8	C5—C4—C3	111.37 (13)
H1—N1—H2	108.9	C5—C4—H9	109.4
C1—N1—H3	112.2	C3—C4—H9	109.4
H1—N1—H3	109.1	C5—C4—H10	109.4
H2—N1—H3	108.2	C3—C4—H10	109.4
N1—C1—C6	109.37 (10)	H9—C4—H10	108.0
N1—C1—C2	110.38 (10)	C4—C5—C6	111.12 (12)
C6—C1—C2	111.97 (10)	C4—C5—H11	109.4
N1—C1—H4	108.3	C6—C5—H11	109.4
C6—C1—H4	108.3	C4—C5—H12	109.4
C2—C1—H4	108.3	C6—C5—H12	109.4
C1—C2—C3	110.31 (11)	H11—C5—H12	108.0
C1—C2—H5	109.6	C1—C6—C5	110.81 (12)
C3—C2—H5	109.6	C1—C6—H13	109.5
C1—C2—H6	109.6	C5—C6—H13	109.5
C3—C2—H6	109.6	C1—C6—H14	109.5
H5—C2—H6	108.1	C5—C6—H14	109.5
C2—C3—C4	111.14 (13)	H13—C6—H14	108.1
C2—C3—H7	109.4	O2—N2—O3	121.20 (10)
C4—C3—H7	109.4	O2—N2—O1	120.99 (10)
C2—C3—H8	109.4	O3—N2—O1	117.79 (10)
C4—C3—H8	109.4		
			
N1—C1—C2—C3	178.21 (11)	C3—C4—C5—C6	−55.5 (2)
C6—C1—C2—C3	56.11 (15)	N1—C1—C6—C5	−178.51 (11)
C1—C2—C3—C4	−55.73 (16)	C2—C1—C6—C5	−55.83 (15)
C2—C3—C4—C5	56.08 (19)	C4—C5—C6—C1	55.08 (18)

### Hydrogen-bond geometry (Å, º)

**Table:**

*D*—H···*A*	*D*—H	H···*A*	*D*···*A*	*D*—H···*A*
N1—H1···O1^i^	0.97	1.89	2.8553 (14)	172
N1—H2···O3^ii^	0.94	1.97	2.9074 (15)	172
N1—H3···O1^iii^	0.85	2.24	2.9880 (15)	148
N1—H3···O3^iii^	0.85	2.28	3.0689 (15)	155

Symmetry codes: (i) −*x*+2, −*y*+1, −*z*+2; (ii) *x*, *y*−1, *z*; (iii) −*x*+2, *y*−1/2, −*z*+3/2.
